# Antimicrobial Activity of Ca-Alginate/Chitosan Nanocomposite Loaded with Camptothecin

**DOI:** 10.3390/polym13203559

**Published:** 2021-10-15

**Authors:** Wafa Al-Gethami, Noha Al-Qasmi

**Affiliations:** Department of Chemistry, College of Science, Taif University, Taif 21944, Saudi Arabia; noha.alqasmi@tu.edu.sa

**Keywords:** calcium-alginate, chitosan, CPT, nanomaterials, antimicrobial effect

## Abstract

The main aim of this study was to prepare antimicrobial nanocomposites consisting of alginate, chitosan, and camptothecin (CPT). CPT-loaded calcium alginate (Ca-Alg_2_) and calcium alginate/chitosan (Ca-Alg_2_-CH) nanomaterials were synthesized and characterized using infrared (IR) spectroscopy, X-ray diffraction (XRD), UV-Vis spectroscopy, and scanning electron microscopy (SEM). The antimicrobial activity and the genetic effects of Ca-Alg_2_/CPT and Ca-Alg_2_-CH/CPT nanomaterials on *Staphylococcus aureus*, *Escherichia coli*, and *Klebsiella pneumonia* were studied. The repetitive element polymerase chain reaction analysis technique was used to assess the changes in the bacterial genetic material due to the processing of the nanomaterials. The results showed the presence of a strong chemical interaction between alginate and chitosan, and CPT was loaded successfully in both Ca-Alg_2_/CPT and Ca-Alg_2_-CH/CPT nanomaterials. Furthermore, the antimicrobial test showed that the Ca-Alg_2_/CPT nanocomposite was susceptible to S. aureus, *E. coli*, and *K. pneumonia*; on the other hand, Ca-Alg_2_-CH/CPT nanocomposite was more susceptible to *E. coli* and *K. pneumonia* and was resistant to *S. aureus.* The results showed that the Ca-Alg_2_/CPT nanocomposite was less efficient than Ca-Alg_2_-CH/CPT nanocomposite in killing Gram-negative treated bacteria. Moreover, results revealed that the PCR analysis revealed a polymorphic banding pattern. This observation provides an excellent guide to the ability of some polymers to induce point mutations in DNA.

## 1. Introduction

Cancer remains a major global health issue in recent years. Scientists have attempted to synthesize novel drug delivery systems (DDSs) to deliver anti-cancer drugs to tumors. Camptothecin (CPT) is an important drug that shows antitumor activity against a variety of tumors such as lung, ovarian, breast, pancreas, and stomach [1]. CPT exists in two forms: a ring-closed lactone form (active against cancer) and a ring-open carboxylate form (inactive against cancer). Under neutral and basic pH conditions, the lactone form transforms to the carboxylate form, which is not only inactive but also toxic [2]. Its poor solubility and poor stability limit its clinical applications. Thus, significant efforts have been made to develop CPT nanocarriers. For example, CPT has been incorporated into organic nanocarriers [3,4], inorganic nanocarriers [4,5], and hybrid nanocarriers [5,6,7].

Although most of these DDSs exhibit many advantages, such as high structural and chemical homogeneity, high drug efficiency, and controlled drug release, some of them suffer from biocompatibility issues, toxicity of some components, interaction with serum proteins, and low drug stability [6,7]. Many studies have focused on designing a novel DDS that distributes the active drug molecule only to the site of action, without affecting healthy organs and tissues.

Biopolymer nanoparticles have attracted considerable attention as vehicles for DDSs. Most biopolymers are polysaccharides and present many advantages such as non-toxicity, hydrophilicity, biocompatibility, and biodegradability in the human body [7,8]. Many of these polysaccharides are bioadhesive because they have hydroxyl, carboxyl, and amino groups that can interact with biological tissues in a non-covalent manner, allowing the material to have a prolonged half-life in the body [7]. Polysaccharides are classified as non-polyelectrolytes and polyelectrolytes, which are either positively or negatively charged [8]. Typical examples of polysaccharides as biopolymers are chitosan, hyaluronan, agar, dextran, dextrin, cellulose, and alginate [8]. Because biopolymers have huge advantages, many researchers have focused on designing DDSs based on biopolymer materials.

Alginate is an anionic natural biopolymer composed of d-mannuronic acid and l-guluronic acid. It is widely used in biomedical applications. For example, Uyen et al. [9] prepared and characterized alginate microsphere-loaded curcumin with an average diameter ranging from 20.2 to 72.3 µm. They studied its antimicrobial activity and found that the microspheres were susceptible to *Staphylococcus aureus* with an inhibition zone diameter of 1.21 cm and were resistant to *Escherichia coli* [9]. 

Alginate has also been used in conjunction with other polymers to improve the performance of drug loading and to enhance its antimicrobial activity. The antimicrobial properties of alginate-derived surfactant (AS) and its metal complexes with cobalt (AS-Co), copper (AS-Cu), and zinc (AS-Zn) were investigated by Tawfik et al. [10], who found that alginate-derived cationic surfactant–zinc, copper, and cobalt complexes showed good antimicrobial activity against Gram-positive and Gram-negative bacteria. They attributed this result to the tendency of metal ions to form complexes with the biological components in the cytoplasm [10]. Venkatesan et al. [11] prepared porous antimicrobial composites consisting of chitosan, alginate, and biosynthesized silver nanoparticles (chitosan-alginate-AgNPs nanocomposites). The pore size of the nanocomposites was 50–500 μm [11]. Antimicrobial activity of the nanocomposites was checked against *E. coli and S. aureus*. It was demonstrated that by adding AgNPs, the antimicrobial property of the chitosan-alginate composite was significantly increased [11]. Thaya et al. [12] prepared chitosan-alginate (CH/ALG) microspheres. They studied the antimicrobial activity of CH/ALG microspheres and observed that increasing the concentration of microspheres resulted in increased inhibition zone sizes against various microbial pathogens [12]. 

The aim of this study was to improve the antimicrobial properties of the Ca-Alg_2_ and Ca-Alg_2_-CH nanocomposites by loading with camptothecin. The prepared nanomaterials were characterized using infrared (IR) spectroscopy, X-ray diffraction (XRD), UV-Vis spectroscopy, and scanning electron microscopy (SEM). The antimicrobial activity and genetic effects of the Ca-Alg_2_/CPT and Ca-Alg_2_-CH/CPT nanomaterials are described.

## 2. Materials and Methods

### 2.1. Materials

Na-Alginate, chitosan, CaCl_2_, HCl, and camptothecin were purchased from Sigma Aldrich and used as purchased. Double-distilled water was used for all solution preparation and rinsing. 

#### 2.1.1. Preparation of Ca-Alg_2_ Nanoparticles

An aqueous solution of Na-Alg (0.3% *wt*/*wt*) was prepared as described in the previous work [13]. To prepare Ca-Alg_2_ nanoparticles, 2 mL of CaCl_2_ (0.3%) was added drop by drop to a 10 mL Na-Alg solution under constant stirring. After the process was complete, the solution allowed for stirring for about 30 min. Then, the suspension was centrifuged at 9000 rpm for 45 min, and the precipitate was washed with distilled water and then placed for drying. 

#### 2.1.2. Preparation of Ca-Alg_2_-CH Nanocomposite

A 0.3% (*w*/*v*) CH solution was prepared using 1% HCl solution. To prepare Ca-Alg_2_-CH nanocomposite, after 30 min of addition of CaCl_2_ (0.3%) to 10 mL Na-Alg, 4 mL of CH solution was slowly added and stirred for 1 h at 500 rpm. The rest of the procedure was the same as that used for the preparation of Ca-Alg_2_ nanoparticles.

#### 2.1.3. CPT Loaded Nanomaterials

To prepare Ca-Alg_2_/CPT and Ca-Alg_2_-CH/CPT nanomaterials, a solution of 10 mg of CPT in 1 mL DMSO was mixed with continuous stirring for 15 min. After that, the 2 mL of 0.3% CaCl_2_ solution was added to this solution with stirring for 15 min. The rest of the procedure was the same as that used for the preparation of Ca-Alg_2_ and Ca-Alg_2_-CH nanomaterials.

#### 2.1.4. Characterization Instrument

The IR spectra of nanomaterials were recorded using UV-Vis spectrometer (Bruker) to demonstrate the preparation of Ca-Alg_2_-CH nanocomposite, and the loading of CPT in the two nanomaterials. The spectroscopic analysis for detection of CPT in nanomaterials were performed using PerkinElmer 750 Lambda UV/VIS/NIR spectrophotometer. The XRD result was measured using scanting XDS2000 powder diffractometer equipped with Cu Kα radiation (λ = 1.540 A°) at 40 kV and 40 mA. The surface morphologies of nanomaterials before and after loading with CPT were obtained using a scanning electron microscope (JEOL-JSM6390LA), the samples sputter-coated with gold prior to imaging. The particles size could be calculated using the Debye–Scherrer equation.

#### 2.1.5. Antimicrobial Activity

##### Bacterial Strains

*Staphylococcus aureus* as positive Gram and *E*. *coli* and *klebsiella penomenas* as negative Gram were used to determine the antibacterial activity. The bacteria were obtained as a kindly gift from Dr. Mohamed M. Hassan at Biology Department, Faculty of Science, Taif University, Saudi Arabia.

##### Repetitive Sequence Analysis of the Genomic DNA

The bacterial genomic DNA of tested bacteria was extracted using the Promega DNA extraction kit (Germany) according to the manufacturer’s instructions. Four primers of rep-PCR were used to determine the changes in genomic DNA of treated bacteria. The primers sequences were as follows: rep-1 (5′-CTACGGCAAGGCGACGCTGACG-3′), rep-2 (5′-GTGGTGGTGGTGGTG-3′).rep-3 (5′-ACACACACACACACACG-3′), and rep-4 (5′-AGAGAGAGAGAGAGAGTT-3′); PCR amplification conditions were carried out according Hassan et al., 2014 [14] without any modification.

##### Statistical Analysis

All of the trails were carried out three times. The connection between the growth turbidity and the concentrations of crude extract was assessed using one-way ANOVA for the three tested strains treated with varied concentrations of Ca Alg_2_/CPT and Ca-Alg_2_-CH/CPT nanomaterials.

## 3. Results and Discussion

### 3.1. Characterization of Ca-Alg_2_/CPT and Ca-Alg_2_-CH/CPT Nanomaterials

**Figure 1:**
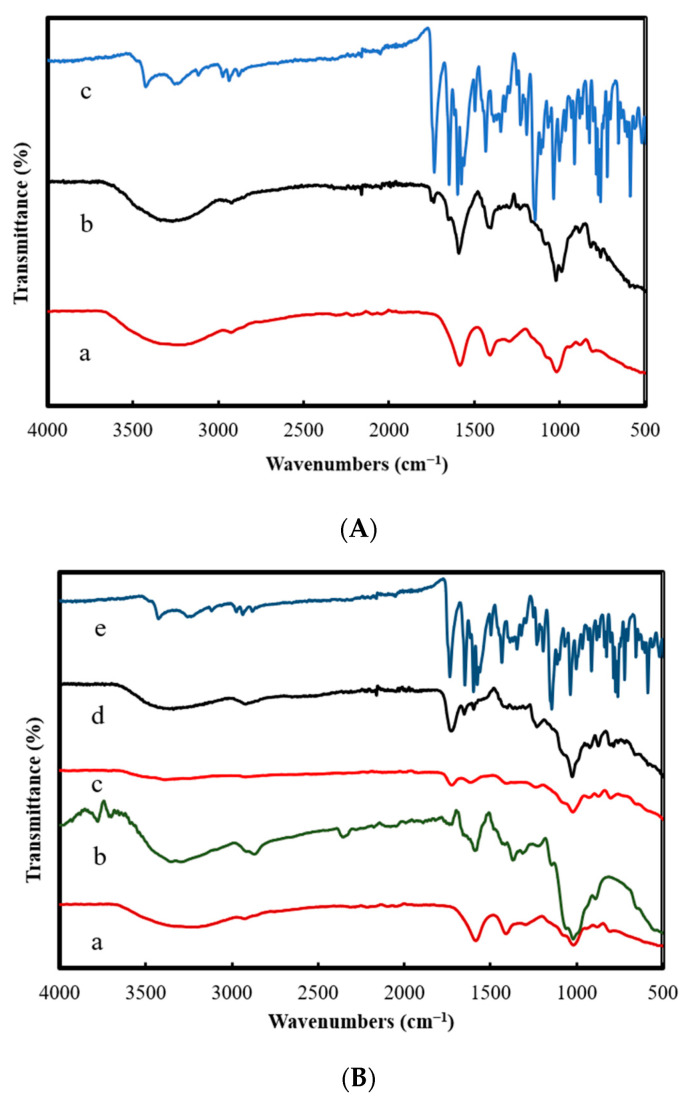
(**A**) IR spectra of Ca-Alg_2_ (a), Ca-Alg_2_/CPT (b), and CPT (c). (**B**) FTIR spectra of Ca-Alg_2_ (a), CH (b), Ca-Alg_2_-CH (c), Ca-Alg_2_-CH/CPT (d), and CPT (e).

Figure 1A shows the IR spectrum (a) of Ca-Alg_2_ nanoparticles. The spectrum shows a C-H stretching vibration at ~2938 cm^−1^ and C-H_2_ bending at ~1416 cm^−1^. The O-H stretching vibration at ~3268 cm^−1^ confirmed the presence of hydrogen bonding of the O-H groups. Finally, the carboxyl groups were observed as asymmetric C=O stretching vibrations at ~1589 cm^−1^ and symmetric C–O stretching vibrations at ~1416 cm^−1^. The band at around 1026 cm^−1^ can be attributed to C–O–C stretching vibration. This is in agreement with a previous report [13]. The spectrum of CPT (c) shows principal peaks at 1650 and 1736 cm^−1^, corresponding to the stretching vibrations of the carbonyl group C=O in the ketone and lactone rings, respectively. The aromatic C–H stretching vibration of amino quinoline was observed at ~3264 cm^−1^, and the peaks appearing at 2977–2885 cm^−1^ represent the stretching vibrations of the CH_3_ group. The peak at ~3430 cm^−1^ can be attributed to hydrogen bonding of the –OH groups. Finally, two peaks at 1599 and 1581 cm^−1^ are attributed to the skeletal vibrations of the phenyl rings [15]. The Ca-Alg_2_/CPT spectrum (b) shows significant differences in bands in comparison with the Ca-Alg_2_ nanoparticle spectrum. The peak attributed to the O–H stretching vibration in the Ca-Alg_2_/CPT nanocomposite spectrum appears narrower than that in the Ca-Alg_2_ nanoparticle spectrum. In addition, C=O stretching vibrations seen at ~1589 cm^−1^ in the Ca-Alg_2_ nanoparticle spectrum were shifted to 1593 cm^−1^ in the Ca-Alg_2_/CPT nanocomposite spectrum, and a new band was observed at ~1740 cm^−1^, which corresponds to the stretching vibrations of the carbonyl group C=O in the lactone ring of CPT, thus indicating the successful loading of CPT on Ca-Alg_2_ nanoparticles.

Figure 1B shows the IR spectrum of CH (b), which shows the common characteristic peaks. The broad band at 3321 cm^−1^ corresponded to –OH (stretching) and amine groups. The absorption band at 2887 cm^−1^ corresponded to –CH (stretching). Carbonyl (C=O) stretching and N–H (bending) vibrations were observed at 1599 and 1375 cm^−1^, respectively. The band at 1024 cm^−1^ was assigned to C–O (stretching) [12,16]. In the spectrum of the Ca-Alg_2_-CH nanocomposite (c), the stretching peak of hydroxyl and amino groups moved to 3383 cm^−1^ and the peak of N–H stretching vibration was shifted to 1407 cm^−1^. In addition, the asymmetrical and symmetrical stretching of –COO– groups shifted to 1725 and 1616 cm^−1^ because of the reaction between sodium alginate and chitosan. The IR spectrum of Ca-Alg_2_-CH/CPT (Figure 1B(d)) shows the characteristic absorption peaks of CPT, suggesting that CPT molecules were conjugated to the polymeric network.

**Figure 2:**
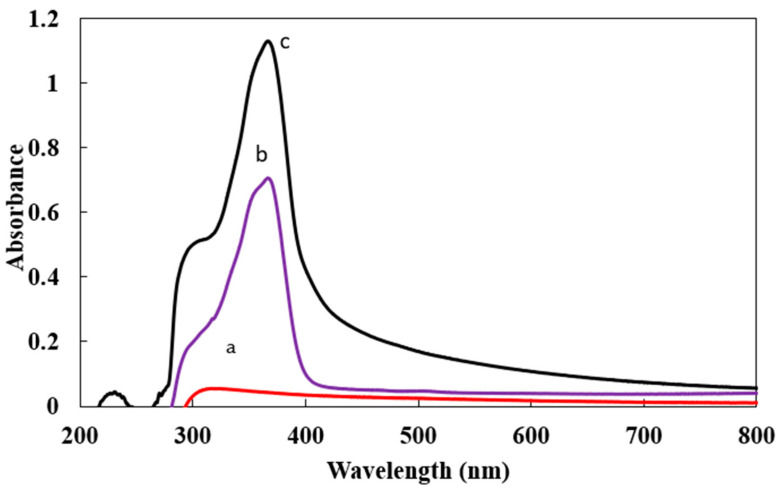
UV visible absorption of (a) Ca-Alg_2_-CH, (b) Ca-Alg_2_-CH/CPT, and (c) Ca-Alg_2_/CPT.

The formation of Ca-Alg_2_/CPT and Ca-Alg_2_-CH/CPT was verified by UV-Vis spectroscopy. Figure 2 displays the UV-Vis spectra of Ca-Alg_2_-CH (a), Ca-Alg_2_-CH/CPT (b), and Ca-Alg_2_/CPT (c). Both Ca-Alg_2_/CPT and Ca-Alg_2_-CH/CPT spectra show a peak at 366 nm, which corresponds to CPT. This indicated the successful loading of CPT in Ca-Alg_2_ and Ca-Alg_2_-CH nanomaterials. As seen, CPT-loaded Ca-Alg_2_ nanoparticles had a higher absorbance than did the Ca-Alg_2_-CH nanocomposite, which is consistent with the SEM results. This can be attributed to the change in the surface morphology of Ca-Alg_2_ after modification by chitosan. 

**Figure 3:**
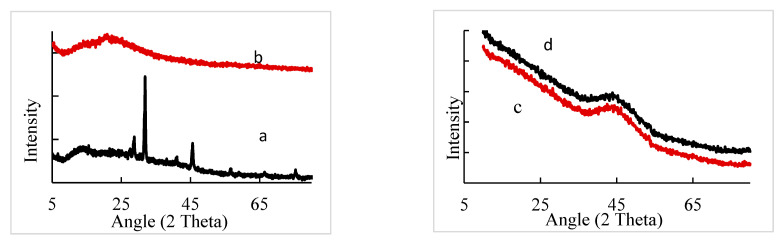
XRD patterns of Ca-Alg_2_ (a), Ca-Alg_2_-CH (b), Ca-Alg_2_-CH/CPT (c), and Ca-Alg_2_/CPT (d).

Figure 3 shows the XRD patterns of Ca-Alg_2_, Ca-Alg_2_-CH, Ca-Alg_2_/CPT, and Ca-Alg_2_-CH/CPT. The Ca-Alg_2_ nanoparticles exhibited two peaks at 2θ = 13.7° and 28.8°, demonstrating the crystalline and amorphous states, respectively [17,18]. For CPT-loaded Ca-Alg_2_ nanoparticles (Figure 3d), a broad diffraction peak centered around 44° was observed. The amorphous nature of the Ca-Alg_2_/CPT nanocomposite indicated that the lattice density of Ca-Alg_2_ nanoparticles decreased after loading CPT [18]. The interaction between chitosan and alginate was confirmed by the XRD pattern shown in Figure 3b. One crystalline diffraction peak was observed at 20.9° [11]. Figure 3c shows the XRD scan of the CPT-loaded Ca-Alg_2_-CH nanocomposite. Most of the sharp peaks attributed to CPT disappeared and only one broad diffraction peak centered again at 44° was observed. On comparing the XRD spectra of Ca-Alg_2_-CH before and after loading CPT, the shift peak position was observed, indicating the interaction between CPT and the Ca-Alg_2_-CH nanocomposite. Obviously, Ca-Alg_2_ nanoparticles showed the highest crystallinity, but the addition of chitosan altered the alignment of the polymer crystal, leading to lower crystallinity. The IR and XRD results prove that CPT was successfully loaded in both Ca-Alg_2_ nanoparticles and Ca-Alg_2_-CH nanocomposite. 

**Figure 4:**
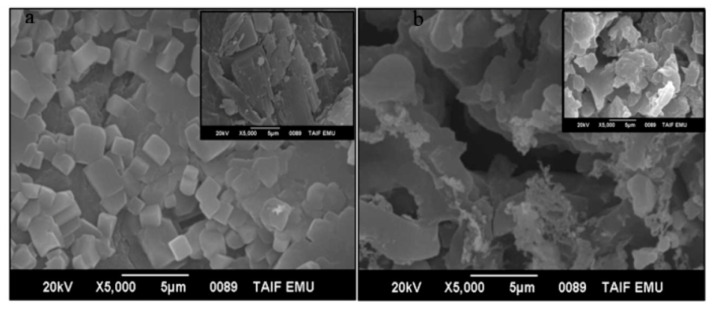
SEM images of (**a**) Ca-Alg_2_ nanoparticles (inserted image of Ca-Alg_2_ after loading CPT), and (**b**) Ca-Alg_2_-CH nanocomposite (inserted image of Ca-Alg_2_-CH after loading CPT).

The SEM images of Ca-Alg_2_, Ca-Alg_2_-CH, Ca-Alg_2_/CPT, and Ca-Alg_2_-CH/CPT are shown in Figure 4. The SEM image of Ca-Alg_2_ nanoparticles (Figure 4a) shows cubic particles with different sizes. After loading CPT in Ca-Alg_2_, the surface of the Ca-Alg_2_/CPT nanocomposite, as shown in the inset, became rough and showed agglomeration, which agrees with the XRD results. It is apparent from the SEM image that the Ca-Alg_2_-CH nanocomposite, as shown in Figure 4b, is considerably different from Ca-Alg_2_. The nanocomposite had a highly porous surface. This change in the surface morphology indicated the modification of Ca-Alg_2_ nanoparticles by chitosan [19].

### 3.2. Antimicrobial Effects of Ca-Alg_2_/CPT and Ca-Alg_2_-CH/CPT Nanomaterials

**Figure 5:**
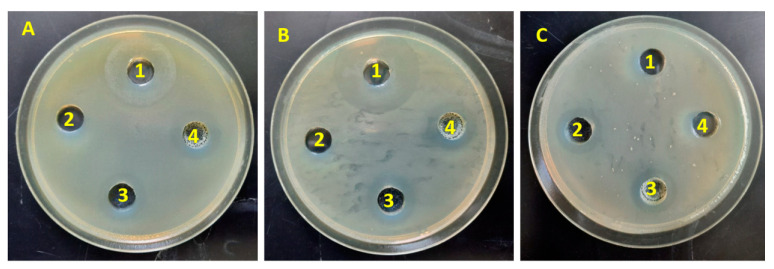
Inhibition zone of different Polymer types (Ca-Alg_2_/CPT and Ca-Alg_2_-CH/CPT) of against (**A**)) *Staphylococcus aureus*, (**B**) *E. coli*, and (**C**) *klebsiella penomenas*. 1 = 1.2 µg/mL of Streptomycin, 2 = DMSO only, 3 = Ca-Alg_2_/CPT (3 mg/mL) and 4 = Ca-Alg_2_-CH/CPT (3 mg/mL).

The antimicrobial activity of two nanomaterials, Ca-Alg_2_/CPT and Ca Alg_2_-CH/CPT, was assessed using the Agar well diffusion method in this work. This approach provides for improved extract diffusion into the media, resulting in more contact with the organisms. The antibacterial activity of DMSO as a solvent at concentrations of 3 mg/mL of Ca-Alg_2_/CPT and Ca-Alg_2_-CH/CPT nanomaterials shows a clear inhibitory zone (in mm diameter). The two nanomaterials were used against three pathogenic organisms, *Staphylococcus aureus* as Gram-positive bacteria and *Escherichia coli* and *klebsiella penomenas* as Gram-negative bacteria. The data are presented in Figure 5 and Table 1. The previous studies [9,20] reported that the antimicrobial activity of Ca-Alg_2_ was relatively poor. When CPT loaded Ca-Alg_2_ nanocomposite (Ca-Alg_2_/CPT), the nanocomposite showed a clear inhibition of the three tests bacteria. The result showed that the Ca-Alg_2_/CPT nanocomposite at final concentrations of 3 mg/mL for the two volumes (200 and 300 µL) were active against the three types of microorganism. The maximum inhibition zone was seen against *E. coli* (11 mm). This observation suggests that Ca-Alg_2_/CPT nanocomposite was more susceptible to *S. aureus*, *E. coli*, and *k. penomenas.* The results also indicated that Ca-Alg_2_-CH/CPT nanocomposite was active against three test strains at final concentrations of 3 mg/mL for the two volumes (200 and 300 µL). According to Venkatesan et al. (2017) [11], chitosan–alginate composite had no inhibitory action against any bacterium, both Gram-positive and Gram-negative. The polyelectrolyte complex system is formed when positive amine groups of chitosan interact with negative carboxylic groups of alginate. This reduces the number of positive charges available to interact with bacterial cell walls. On the other hand, when CPT loaded Ca-Alg_2_-CH nanocomposite (Ca-Alg_2_-CH/CPT), the nanocomposite displayed a larger zone of inhibition on Gram-negative bacteria *E. coli and K. pneumonia*. The maximum activity of Ca-Alg_2_-CH/CPT nanocomposite was seen against *E. coli* (12 mm). Ca-Alg_2_-CH/CPT nanocomposite had a large inhibition zone against *E. coli and K. pneumonia* bacteria than Ca-Alg_2_/CPT nanocomposite, suggesting that the positive amine groups of chitosan that are not engaged with carboxylic groups may react with the cell membrane of negatively charged bacteria, preventing bacterial growth. Moreover, as shown in Table 1, the inhibition zone diameter for both nanocomposites against *S. aureus* bacteria was (9 mm) at final concentration. These finding suggested that *S. aureus* was the most resistant organisms of the Ca-Alg_2_-CH/CPT nanocomposite. Overall, the antimicrobial activity of both nanomaterials was improved by loading CPT; however, CPT’s antimicrobial activity remains unclear [21]. 

### 3.3. The Genetic Effects of the Ca-Alg_2_/CPT and Ca-Alg_2_-CH/CPT Nanocomposites

**Figure 6:**
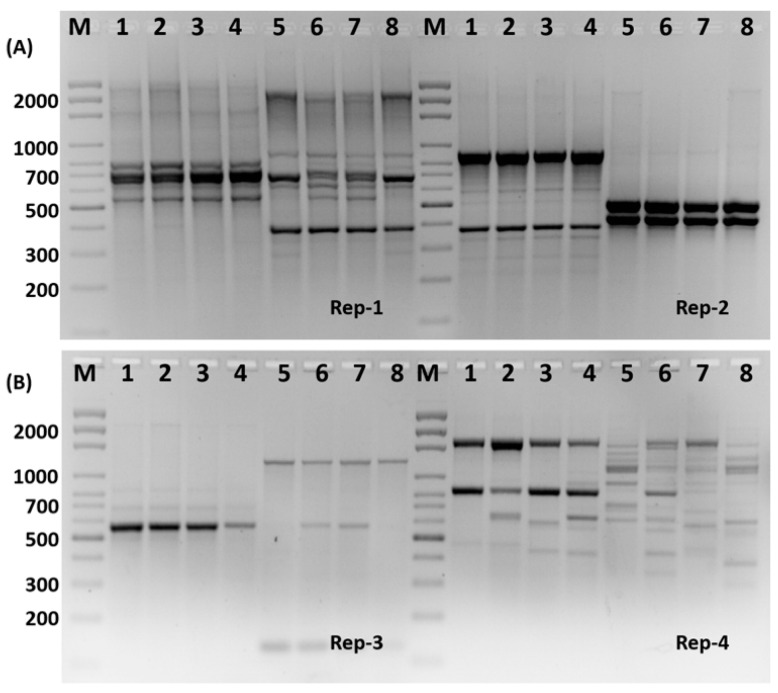
Rep-PCR profile of treated *Staphylococcus aureus* (**A**) and *klebsiella penomenas* (**B**) strains generated with Rep primer No. 1–4 using different concentration of Ca-Alg_2_-CH/CPT nanocomposites (2–4), 1 = untreated strains. M: is 100 bp DNA ladder, the concentrations of Ca-Alg_2_-CH/CPT nanocomposites 1, 2, and 3 mg/mL, respectively.

The changes in the bacterial genetic material due to the treatment of the polymers were evaluated using Rep-PCR analytes in order to explore the stability of the genetic material, at the molecular level, in the treated bacteria as a result of the antimicrobial activities of the different concentrations of Ca-Alg_2_/CPT and Ca-Alg_2_-CH/CPT nanomaterials (100, 200, and 300 µL) of 3 mg/mL polymer. When comparing the electrophoretic products of PCR for the treated *Staphylococcus aureus* and *Klebsiella penomenas* bacteria strains to those of untreated bacteria, the Rep-PCR results revealed a large number of polymorphic numbers of the genetic bands (Figure 6a,b). Figure 6 illustrates that the highest number of polymorphic bands among treated *klebsiella penomenas* (1–4) was generated in reaction with the primer rep-1 (8 monomorphic bands), and this represented 100% of the total bands. Next, rep-2 primer was the second number of the highest number of polymorphic bands (9), and polymorphic bands were obtained with primer rep-4 with molecular bands 600 and 650 bp compared with the untreated strain (control). Interestingly, when we use Rep-PCR as a primer with the genome of the treated *Staphylococcus aureus* strain (5–8) generated in reaction with the primer rep-1, we can obtain a unique positive band at a molecular weight of 600 bp (Figure 6a). Surprisingly, the clear band at the molecular weight of 590 bp was absent in the control *Staphylococcus aureus* strain (untreated bacteria) and detected in the treated *Staphylococcus aureus* strain (Figure 6b—rep-3). Additionally, the bands with molecular weight about 200, 300, 400, 900, and 1200 bp were shown as a positive unique band in the treated strain (Figure 6b—rep-4).

Rep-PCR was used to confirm the genetic effect of the Ca-Alg_2_/CPT and Ca-Alg_2_-CH/CPT nanomaterials as mutagenic agents in our investigation. When comparing untreated bacteria to those treated with different concentrations of Ca-Alg_2_/CPT and Ca-Alg_2_-CH/CPT nanomaterials, the results revealed a polymorphic banding pattern (Figure 6). This finding supports the theory that certain polymers can cause point mutations by deleting at least one nucleotide, as seen by the removal of many genetic bands and changes in primer matching sites when compared to untreated bacteria. These findings point to molecular changes such as deletion or frame shift mutations in one or more loci, which impact gene expression and, as a result, disrupt DNA and protein synthesis biochemical pathways. These results are consistent with the results obtained [22,23].

## 4. Conclusions

Ca-Alg_2_/CPT and Ca-Alg_2_-CH/CPT nanomaterials were successfully prepared, and their antimicrobial properties were determined. UV-Vis spectroscopy and IR spectroscopy results confirmed the presence of CPT in Ca-Alg_2_ and Ca-Alg_2_-CH nanomaterials. SEM results revealed that the surface morphology of the nanomaterials changed after loading with CPT. The antimicrobial study results showed that *S. aureus*, *E. coli*, and *Klebsiella pneumonia* were more susceptible to the Ca-Alg_2_/CPT nanocomposite. The antibacterial action of the nanocomposite was enhanced by the presence of CPT. After modified Ca-Alg_2_ with chitosan and loaded with CPT drug, Ca-Alg_2_-CH/CPT nanocomposite was more susceptible to *E. coli* and *k. penomenas* and was resistant to *S. aureus*. The inhibition zone increased against *E. coli* and *K. pneumonia* bacteria, but there was no increase in the inhibition zone against *S. aureus.* bacteria. Increasing the zone of inhibition may refer to the interaction between the positive amine groups in chitosan with negative charge of the bacterial cell wall. Furthermore, the PCR study revealed a polymorphic banding pattern, which is a good indicator of the ability of some polymers to induce point mutations in DNA. The results presented here may provide some basis for the application of Ca-Alg_2_ and Ca-Alg_2_-CH nanomaterials as a delivery system for CPT.

## Figures and Tables

**Table 1:** Diameter of inhibition zone (DIZ) in mm of three bacterial strains caused by Ca-Alg_2_/CPT and Ca-Alg_2_-CH/CPT nanomaterials.

Strains	Streptomycin	Ca-Alg_2_/CPT (3 mg/mL)		Ca-Alg_2_-CH/CPT (3 mg/mL)	
		200 µL	300 µL	200 µL	300 µL
*S. aureus*	14	7	9	8	9
*E. coli*	13	9	11	10	12
*K. penomenas*	0	8	9	8	10

## Data Availability

Data is contained within the article.
